# Comparison of adenoma detection in different colorectal segments between deep-sedated and unsedated colonoscopy

**DOI:** 10.1038/s41598-022-19468-y

**Published:** 2022-09-12

**Authors:** Yue Sui, Qing Wang, Hai-Hua Chen, Jun-Hui Lu, Qing Wen, Zhen-Zhen Wang, Guan-Feng Wang, Hui Jia, Tao Xiao, Na-Ping Wang, Jun-Lian Hao, Yi-Ping Zhang, Feng-Zhen Cao, Xiao-Peng Wu, Xing Chen

**Affiliations:** 1 Shanxi Medical University, Taiyuan, Shanxi China; 2 First Hospital of Shanxi Medical University, No.85 of South Xinjian Road, Yingze District, Taiyuan, 030000 Shanxi China; 3 The Second People’s Hospital of Datong, Datong, Shanxi China; 4 Ordos Mongolian Medical Hospital, Ordos, Inner Mongolia China; 5 Shanxi Tumor Hospital, Taiyuan, Shanxi China; 6 Yanhu District Branch, The First Hospital of Shanxi Medical University, Yuncheng, Shanxi China; 7 Xiaoyi Traditional Chinese Medicine Hospital, Xiaoyi, Shanxi China; 8 Datong Shoujia Digestive Disease Hospital, Datong, Shanxi China; 9 Ordos Kangning Physical Examination Center, Ordos, Inner Mongolia China; 10 Lvliang Traditional Chinese Medicine Hospital, Lvliang, Shanxi China

**Keywords:** Medical research, Colonoscopy

## Abstract

To investigate if deep-sedated colonoscopy affects adenoma detection in certain colorectal segment. Review of colonoscopy reports, electronic images and medical records of individuals underwent screening colonoscopy with or without propofol sedation between October 2020 and March 2021 from seven hospitals in China. A total of 4500 individuals were analyzed. There was no significant difference in ADR between deep-sedated colonoscopy and unsedated colonoscopy [45.4% vs. 46.3%, *P* > 0.05]. The APP of deep-sedated colonoscopy was lower than unsedated colonoscopy (1.76 ± 0.81 vs. 2.00 ± 1.30, *P* < 0.05). Both average number of adenomas and luminal distention score of splenic flexure and descending colon were lower in deep-sedated colonoscopy (*P* < 0.05), and average number of adenomas was positively correlated with an improved distension score in splenic flexure and descending colon (splenic flexure r = 0.031, *P* < 0.05; descending colon r = 0.312, *P* < 0.05). Linear regression model showed deep-sedated colonoscopy significantly affected luminal distention of splenic flexure and descending colon as well as average number of adenomas detected in splenic flexure (*P* < 0.05). Deep-sedated colonoscopy decreased adenoma detection in splenic flexure and the luminal distention of splenic flexure and descending colon compared with unsedated colonoscopy.

## Introduction

According to the Global Cancer Report 2020, colorectal cancer (CRC)ranks third in incidence and second in mortality of cancer-related diseases worldwide^1^. Colonoscopy is currently considered to be the gold standard for detecting CRC and precancerous lesions^2–5^. However, sometimes CRC is detected within surveillance interval after negative colonoscopy, which is called post-colonoscopy colorectal cancer (PCCRC)^6^. High-quality baseline colonoscopy is of great importance in preventing PCCRC^7,8^. Current colonoscopy quality indicators include adenoma detection rate (ADR), cecal intubation rate (CIR), polypectomy rate, colonoscope withdrawal time, quality of bowel preparation and adverse or unplanned events after colonoscopy ^5,9–11^.

Over the past decade, sedated colonoscopy has been increasing substantially all over the world^2,12–17^. One reason is that adequate sedation contributes to better patient experience in terms of greater patient cooperation, less patient memory of discomfort, reduction in reported pain and increase in patient tolerance of the procedure which encourage more people to have a CRC screening. However, the benefit of sedation on colonoscopy quality is still controversial^2,14,15,18–21^. Deep sedation and conscious sedation are the two most common types of patient comfort management, while in China conscious sedation is barely used in routine colonoscopy. Individuals are given two choices, deep sedation or no sedation, when booking an appointment. Colonoscopy was almost conducted entirely with the patient in the left lateral position during deep sedation because it is inconvenient to make the deep-sedated patient change position, and supine position during deep sedation may increase respiratory movements, choking rates and other respiratory problems. Both splenic flexure and descending colon are difficult to be completely visualized endoscopically in the left lateral position comparing with the right lateral position because of inadequate luminal distention. We presume that deep-sedated colonoscopy might impair adenoma detection in these segments and conducted the present study to compare the luminal distention and number of adenomas detected in different colorectal segments between deep-sedated colonoscopy and unsedated colonoscopy.

## Methods

### Study design

The present study involved seven endoscopy centers chosen from Dr. Chen Xing Workstation in China, where both sedation practice during endoscopy and endoscopy performance have been standardized with high quality. Deep-sedated colonoscopy is routinely performed in all the participating centers without substantial difference in proportion of deep-sedated to unsedated procedures, and all endoscopists performed both procedures consecutively in daily practice. Inclusion criteria were individuals aged 35–60 years who first underwent screening colonoscopy with deep sedation or without sedation from October 2020 to March 2021, colonoscopy performed by endoscopists from gastroenterology department with at least 10 years’ experience, perform at least 2000 colonoscopy every year, and ADR no less than 40% in the past three years. Exclusion criteria were individuals with polyposis syndrome, inflammatory bowel disease, CRC, history of abdominal or pelvic surgery, poor bowel preparation (Boston Bowel Preparation scale, BBPS < six points or any segmental score < two points), withdrawal time less than six minutes and incomplete data. The participating hospitals were Shanxi Provincial Cancer Hospital, The Second People’s Hospital of Datong, Datong Shoujia Digestive Disease Hospital, Xiaoyi Traditional Chinese Medicine Hospital, Lvliang Traditional Chinese Medicine Hospital, Ordos Kangning Physical Examination Center and The First Hospital of Shanxi Medical University, Yanhu District Branch. The study protocol was approved by the Chinese Ethics Committee of Registering Clinical Trials (IRB number ChiECRCT20210467). The informed consents were obtained from all individuals before they underwent colonoscopy. The necessity for written informed consent in the present study was waived due to the retrospective nature. The study was performed in accordance with the principles of the Declaration of Helsinki.

Each individual underwent bowel preparation in accordance with local practice. At all the above centers, no antispasmodic medication was administered before or during the procedure. Propofol (AstraZeneca Italy, Caponago, Italy) sedation was administered by experienced anesthesiologists. Colonoscopy was performed using high-definition colonoscopes (CF-HQ290I, CF-H260AI, Olympus, Japan; BL-7000, Fujifilm, Japan) without any auxiliary device. Individuals who underwent deep-sedated colonoscopy were kept in the left lateral position throughout the procedure, and those who underwent unsedated colonoscopy took dynamic position changes during withdrawal. Dynamic position changes were as following: supine position for the cecum, ascending colon, hepatic flexure (left lateral position when necessary) and transverse colon; right lateral position (or 30° to the right) for splenic flexure and descending colon; left lateral position for sigmoid colon and rectum^22^. Cecal intubation was confirmed by the observation of cecal landmarks. The location, size and morphology of any polyp found during the procedure were recorded in colonoscopy reports. When a polyp was detected, a biopsy was taken or the polyp was removed and placed in a separate bottle and sent for histopathological examination. Data including age, sex, height, weight, family history of CRC, withdrawal time, BBPS, examination results and luminal distention score were collected from medical records, colonoscopy reports and electronic images. Two experienced endoscopists who were trained for awarding the luminal distension score performed data extraction, and they were blinded to the sedation type.

### Study outcome measures

The primary outcome was the average number of adenomas detected in each colorectal segment (cecum + ascending colon, hepatic flexure, transverse colon, splenic flexure, descending colon, sigmoid colon + rectum). The secondary outcomes were ADR, adenomas per positive patient (APP) and luminal distention score of each colorectal segment.

### Definition

ADR was defined as the proportion of patients with at least one adenoma detected. APP was defined as the average number of adenomas detected in positive patients. A distension score was defined by using a previously validated 5-point scale as follows: 1, total collapse; 2, collapse with view < 2 haustral folds into the distance; 3, some proximal collapse only with “crinkling” of folds; 4, widely distended, distal collapse at limit of vision; and 5, widely distended, no distal collapse to limit of vision^23^.

### Statistical analysis

SPSS version 24.0 (SPSS Inc., Chicago, IL, USA) was used for the data analysis. Exploratory data analysis and Shapiro–Wilk tests were performed to determine the normality of the data distribution. Normally distributed continuous data are expressed as mean ± standard deviation (SD). Categorical variables were presented as counts and percentage. Continuous variables were compared using Student’s t-tests. Categorical variables were compared using the Pearson χ^2^ test. The correlation between luminal distention score and average number of adenomas detected in each colorectal segment was analyzed using Pearson’s correlation analysis. Linear regression model was used to adjust the influence of age, sex, BMI, family history of CRC, bowel preparation quality and withdrawal time. A probability (*p*) value of < 0.05 was considered statistically significant, and all tests were two-tailed.

## Results

### Baseline characteristics

**Figure 1:**
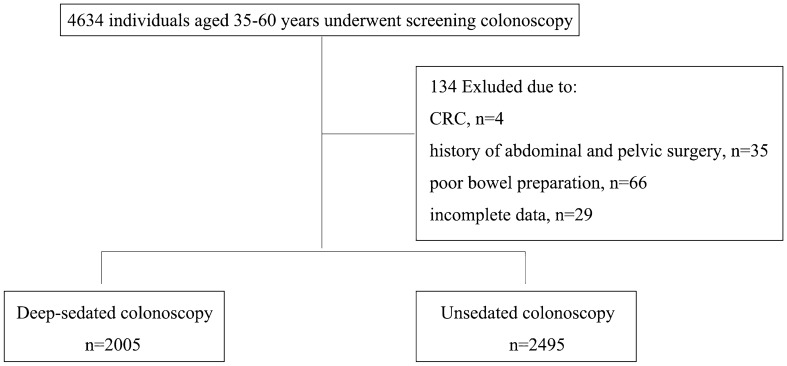
Study subject flow chart.

As shown in Fig. 1, a total of 4634 individuals were included, and 134 cases were excluded for the following reasons: 4 cases with CRC, 35 cases with history of abdominal and pelvic surgery, 66 cases with poor bowel preparation and 29 cases with incomplete data. A total of 4500 individuals were finally analyzed, 2005 in the deep-sedated group and 2495 in the unsedated group.

As shown in Table 1, there was no significant difference between the two groups in terms of age, sex, body mass index (BMI), family history of CRC, withdrawal time and BBPS (*P* > 0.05). Cecal intubation rate was 100% in both groups.

**Table 1:** Comparison of baseline characteristics between deep-sedated group and unsedated group.

	Deep-sedated group (*n* = 2005)	Unsedated group (*n* = 2495)	*t*/χ^2^	*P*
Age (years)	50.30 ± 8.83	50.08 ± 9.14	0.825	0.409
Male/female	1013/992	1317/1178	2.278	0.131
BMI (kg/m^2^)	24.95 ± 2.98	24.99 ± 2.96	0.415	0.678
Family history of CRC [*n*(%)]	185 (9.2)	204 (8.2)	1.554	0.213
Withdrawal time (s)	598.86 ± 81.05	600.10 ± 79.14	0.514	0.608
BBPS	7.87 ± 0.831	7.84 ± 0.813	1.363	0.173

*BMI* body mass index, *CRC* colorectal cancer, *BBPS* boston bowel preparation scale.

### Adenoma detection and luminal distention

1606 adenomas were detected in 910 patients in the deep-sedated group, and 2308 adenomas were detected in 1154 patients in the unsedated group. ADR of the deep-sedated group and the unsedated group were 45.4% (910/2005) and 46.3% (1154/2495) respectively. There was no significant difference in ADR between the two groups (*P* > 0.05). APP of the deep-sedated group and the unsedated group were 1.76 ± 0.81 and 2.00 ± 1.30 respectively. APP of the deep-sedated group was significantly lower than the unsedated group (*P* < 0.05). (Table 2).

**Table 2:** Comparison of ADR and APP between deep-sedated group and unsedated group.

	Deep-sedated group (*n* = 2005)	Unsedated group (*n* = 2495)	χ^2^/*t*	*P*
ADR	45.4% (910/2005)	46.3% (1154/2495)	0.368	0.544
APP	1.76 ± 0.81	2.00 ± 1.30	− 5.005	< 0.05

*ADR* adenoma detection rate, *APP* adenomas per positive patient.

As shown in Table 3, when stratified by different colorectal segments, luminal distention score of splenic flexure in the deep-sedated group and unsedated group were 3.75 ± 1.60 and 4.37 ± 0.66 respectively; luminal distention score of descending colon in the deep-sedated group and unsedated group were 4.25 ± 1.18 and 4.48 ± 0.50 respectively. Luminal distention scores of splenic flexure and descending colon were both significantly lower in the deep-sedated group (*P* < 0.05). Age, sex, BMI, family history of CRC, BBPS and withdrawal time were taken into linear regression model, after adjusting the influence of control variables, deep-sedated colonoscopy significantly affected the luminal distention scores in splenic flexure and descending colon (*P* < 0.05), other colorectal segments were not significantly influenced (*P* > 0.05) (shown in Supplementary Table S1).

**Table 3:** Comparison of luminal distention scores between deep-sedated group and unsedated group.

	Deep-sedated group (*n* = 2005)	Unsedated group (*n* = 2495)	*t*	*P*
Cecum + ascending colon	4.42 ± 0.59	4.39 ± 0.66	1.824	0.068
Hepatic flexure	4.50 ± 0.50	4.49 ± 0.50	0.351	0.725
Transverse colon	4.34 ± 0.65	4.37 ± 0.66	− 1.764	0.078
Splenic flexure	3.75 ± 1.60	4.37 ± 0.66	− 16.097	< 0.05
Descending colon	4.25 ± 1.18	4.48 ± 0.50	− 8.044	< 0.05
Sigmoid colon + rectum	4.26 ± 0.70	4.27 ± 0.71	− 0.431	0.667

As shown in Table 4, the average number of adenomas detected in splenic flexure in the deep-sedated group and unsedated group were 0.01 ± 0.10 and 0.02 ± 0.15 respectively. The average number of adenomas detected in descending colon in the deep-sedated group and unsedated group were 0.16 ± 0.37 and 0.24 ± 0.59 respectively. The average number of adenomas detected in both splenic flexure and descending colon were significantly lower in the deep-sedated group (*P* < 0.05). Linear regression model showed deep-sedated colonoscopy significantly affected average number of adenomas detected in the splenic flexure (*P* < 0.05), other colorectal segments were not significantly influenced (*P* > 0.05) (shown in Supplementary Table S2).

**Table 4:** Comparison of average number of adenomas detected in different colorectal segments.

	Deep-sedated group (*n* = 2005)	Unsedated group (*n* = 2495)	*t*	*P*
Cecum + ascending colon	0.11 ± 0.31	0.12 ± 0.35	− 1.010	0.313
Hepatic flexure	0.03 ± 0.20	0.03 ± 0.18	− 0.113	0.910
Transverse colon	0.12 ± 0.33	0.13 ± 0.37	− 1.157	0.247
Splenic flexure	0.01 ± 0.10	0.02 ± 0.15	− 3.455	0.001
Descending colon	0.16 ± 0.37	0.24 ± 0.59	− 5.339	< 0.05
Sigmoid colon + rectum	0.37 ± 0.48	0.38 ± 0.59	− 0.723	0.470

As shown in Table 5, the average number of adenomas detected in splenic flexure and descending colon demonstrated a positive correlation with luminal distention score (splenic flexure *r* = 0.031, *P* < 0.05; descending colon *r* = 0.312, *P* < 0.05). The correlation was not significant in other colorectal segments (*P* > 0.05).

**Table 5:** Correlation between adenoma detection and luminal distention in different segments.

	*r*	*P*
Cecum + ascending colon	0.012	0.436
Hepatic flexure	− 0.001	0.895
Transverse colon	0.000	0.996
Splenic flexure	0.031	0.039
Descending colon	0.312	< 0.05
Sigmoid colon + rectum	− 0.014	0.336

## Discussion

The present study confirmed that luminal distention scores were significantly lower in both the splenic flexure and descending colon in deep-sedated colonoscopy compared with unsedated colonoscopy. In accordance with luminal distention score, the average number of adenomas detected in splenic flexure and descending colon in the deep-sedated group were lower than the unsedated group. There was a significant positive correlation between the average number of adenomas detected and luminal distention score in the splenic flexure and descending colon. The factors affecting adenoma detection are complex, including withdrawal time, observation of the back side of the wrinkled fold, bowel cleanliness and luminal distention. Changing position during colonoscopy is one of the easiest ways to improve the colonoscopy quality^24^ which could remove liquid from the area to be observed, place the bowel segment at the highest location in the abdominal cavity, open the sharp angle at the folds, improve the luminal distention with a small amount of air insufflation^25^. The use of position changes during the withdrawal phase of colonoscopy was generated from the experience of radiologists with barium enemas, facilitating adequate distension of the colon and movement of excess fluid away from the colonic area of interest^25^. Deep-sedated colonoscopy limits the use of this evidence based effective practice. Previous studies comparing the quality metrics of deep-sedated colonoscopy and conscious colonoscopy barely mentioned whether they took position-change into account in their study design^2,14,18,26,27^. The results in the present study were consistent with previous studies, which reported a significant increase in the number of detected adenomas in transverse colon and left colon using a position-change method^3,4,24,28–30^. However, the position change process performed in the present study was different from the method that Seung-Woo Lee et al.^28^ used as follows: cecum, ascending colon, and hepatic flexure: left lateral position; transverse colon: supine position; splenic flexure, descending colon, sigmoid colon, and rectum: right lateral position. The results from the present study showed adequacy luminal distention and high ADR when cecum, ascending colon, and hepatic flexure were observed in supine position, as well as sigmoid colon and rectum being observed in left lateral position. There seems no significant difference in luminal distention between the two processes, which needs to be confirmed in further study.

Several previous studies demonstrated that the use of sedation (regardless of type) during colonoscopy increased ADR^2,14,18,26,27^. However, ADR only indicated that at least one adenoma was detected, but the total number of adenoma detected in index colonoscopy is a key factor in making appropriate surveillance interval. Yutaka Okagawa et al.^28^ conducted a research to find out the association between second surveillance colonoscopy and the former results by dividing the patients into three risk levels according to the number of adenoma and the detection of advanced adenoma, and demonstrated that patients with high-risk findings on first surveillance colonoscopy should have second surveillance colonoscopy performed at shorter intervals. As shown in the present study, although there was no significant difference in ADR between two groups, APP of the unsedated group was significantly higher than the deep-sedated group, which meant that more adenomas were detected per patient in unsedated colonoscopy, leading to higher risk stratification and shorter surveillance interval.

The overall CIR in the present study was 100%, which was much higher than reported in previous studies. There might be bias in the definition of cecal intubation which was defined as the observation of cecal landmarks. A more reasonable definition should be that tip of the colonoscope touched the appendicular orifice and could be moved freely in the cecum. Unfortunately, the retrospective nature of the study limited the confirmation of cecal intubation according to the latter. Additionally, conditions affecting colorectal luminal distension and adenoma detection were excluded from the study, such history of abdominal or pelvic surgery, CRC and poor bowel preparation, which also contributed to high CIR.

Sedative and analgesic drugs are commonly used to improve the patient experience of colonoscopy, while most experts in the field agree that experienced endoscopists with optimal techniques require minimal or no sedation because they can maneuver the colon without causing pain and discomfort to patients, leading to faster patient recovery and negating the putative benefits of propofol^15,31^. All endoscopists performing colonoscopy should be able to complete colonoscopy safely and effectively (per accepted benchmarks) using moderate sedation or less, and endoscopists unable to do so should undergo additional training^32^. Institutions should not mandate the use of deep sedation for routine colonoscopy^33^. However, generalized conclusions cannot be made since decisions on whether to use sedation are influenced by differences in the sociocultural backgrounds of countries and regions, patient expectations, cost effectiveness, and facility conditions. At a minimum, patients must be fully informed about the risks and benefits of sedation.

This is the first large-scale multicenter study evaluating the quality of deep-sedated colonoscopy in terms of number of adenomas detected in each colorectal segment besides ADR and CIR. Moreover, the present study was designed with high-quality control by only including experienced endoscopists, BBPS no less than six points and any segmental score no less than two points, withdrawal time no less than six minutes, eliminating several confounding factors found in prior studies. However, the retrospective nature of the study lends considerable limitation to the study. First, the benefits seen in this study should ideally be confirmed in a large, multicenter, randomized, parallel-group study; however, randomized allocation of participants to groups with or without deep sedation might give rise to ethical problems. Second, individual variation of colonoscopists was not analyzed in the present study. The colonoscopy included were all performed by experienced endoscopists, so such a conclusion might not be applied to trainee endoscopists. Third, individuals included in the study were between 35 and 60 years old, who cannot represent the CRC screening population. While in China, where medical resource is limited, the main form of screening is opportunistic screening rather than mass screening. The vast majority of opportunistic screening population is made up of individuals aged 35–60 years who schedule for colonoscopy as part of routine physical examination. A wider range of population with various indications for colonoscopy will be included in further study. Fourth, several different types of colonoscope were used in the study which might have some influence on lesion detection. BL-7000 and CF-HQ290I have a viewing range of 170° in regular focal length, while the viewing range of CF-H260AI is 140°. The depth of field of BL-7000, CF-HQ290I, CF-H260AI is 2–100 mm, 9–100 mm and 5–100 mm respectively. CF-HQ290I and CF-H260AI have different image-enhanced model from BL-7000. Differences inevitably exist between different types of colonoscope, whether or not these differences have significant influence on adenoma detection need to be investigated in further study.

In conclusion, although the ADR was high, deep-sedated colonoscopy decreased luminal distention of splenic flexure and descending colon and affected adenoma detection in splenic flexure compared with unsedated colonoscopy. Colonoscopy performed without sedation or conscious sedation which provides patient comfort without generally impeding their ability to change position might be considered as routine practice.

## Supplementary Information

Supplementary Information.

## Data Availability

The datasets generated and/or analyzed during the current study are available in the Science Data Bank repository, https://www.scidb.cn/anonymous/YmVRRkJq.

## Abbreviations

ADR: Adenoma detection rate
APP: Adenomas per positive patient
