# Supplementary material for: Comparison of adenoma detection in different colorectal segments between deep-sedated and unsedated colonoscopy
Source: Sci Rep. 2022 Sep 12;12:15356. doi: 10.1038/s41598-022-19468-y (PMC9468171; doi:10.1038/s41598-022-19468-y)
Supplement: Supplementary file 1 — Supplementary Information. [file 41598_2022_19468_MOESM1_ESM.docx]

**Comparison of adenoma detection in different colorectal segments between deep-sedated and unsedated colonoscopy**

Yue Sui^1^, Qing Wang^1^, Hai-Hua Chen^2^, Jun-Hui Lu^1^, Qing Wen^3^, Zhen-Zhen Wang^3^, Guan-Feng Wang^3^, Hui Jia^4^, Tao Xiao^5^, Na-Ping Wang^6^, Jun-Lian Hao^7^, Yi-Ping Zhang^8^, Feng-Zhen Cao^9^, Xiao-Peng Wu^10^, and Xing Chen*^1,2^

^1^Shanxi Medical University, Taiyuan, Shanxi, China

^2^First Hospital of Shanxi Medical University, Taiyuan, Shanxi, China

^3^The Second People’s Hospital of Datong, Datong, Shanxi, China

^4^Ordos Mongolian Medical Hospital, Ordos, Inner Mongolia, China

^5^Shanxi Tumor Hospital, Taiyuan, Shanxi, China

^6^The First Hospital of Shanxi Medical University, Yanhu District Branch, Yuncheng, Shanxi, China

^7^Xiaoyi Traditional Chinese Medicine Hospital, Xiaoyi, Shanxi, China

^8^Datong Shoujia Digestive Disease Hospital, Datong, Shanxi, China

^9^Ordos Kangning Physical Examination Center, Ordos, Inner Mongolia, China

^10^Lvliang Traditional Chinese Medicine Hospital, Lvliang, Shanxi, China

Supplementary Table S1. Linear regression of luminal distention score in different colorectal segments

|  | Cecum +ascending colon | | Hepatic flexure | | Transverse colon | |
| --- | --- | --- | --- | --- | --- | --- |
|  | M1 | M2 | M3 | M4 | M5 | M6 |
| Age | 0.006 | 0.005 | -0.003 | -0.003 | -0.002 | -0.002 |
| Sex | -0.022 | -0.023 | -0.020 | -0.021 | -0.009 | -0.009 |
| Family history of CRC | -0.045** | -0.046** | 0.025 | 0.025 | -0.024 | -0.023 |
| BMI | -0.003 | -0.003 | -0.019 | -0.019 | 0.006 | 0.006 |
| Withdrawal time | -0.005 | -0.005 | -0.007 | -0.007 | 0.001 | 0.001 |
| BBPS | 0.018 | 0.017 | 0.009 | 0.009 | 0.016 | 0.016 |
| Deep-sedated colonoscopy |  | -0.024 |  | -0.005 |  | 0.026 |
| R^2^ | 0.003 | 0.004 | 0.002 | 0.000 | 0.001 | 0.001 |
| △R^2^ | 0.003 | 0.001 | 0.002 | 0.000 | 0.001 | 0.001 |
| F | 2.205* | 2.269* | 1.162 | 1.012 | 0.701 | 1.033 |
| △F | 2.205* | 2.646 | 1.162 | 0.113 | 0.701 | 3.023 |

BMI, body mass index; CRC, colorectal cancer; BBPS, Boston Bowel Preparation scale.

**P* < 0.05，***P* < 0.01，****P* < 0.001

|  | Splenic flexure | | Descending colon | | Sigmoid colon +Rectum | |
| --- | --- | --- | --- | --- | --- | --- |
|  | M7 | M8 | M9 | M10 | M11 | M12 |
| Age | 0.016 | 0.019 | 0.006 | 0.007 | -0.002 | -0.002 |
| Sex | 0.005 | 0.011 | 0.009 | 0.012 | -0.009 | -0.009 |
| Family history of CRC | -0.015 | -0.010 | -0.015 | -0.012 | -0.011 | -0.011 |
| BMI | -0.011 | -0.013 | -0.011 | -0.012 | -0.001 | -0.001 |
| Withdrawal time | -0.028 | -0.026 | -0.005 | -0.004 | -0.005 | -0.005 |
| BBPS | -0.009 | -0.004 | 0.003 | 0.006 | 0.012 | 0.012 |
| Deep-sedated colonoscopy |  | 0.251*** |  | 0.129*** |  | 0.006 |
| R^2^ | 0.002 | 0.065 | 0.001 | 0.017 | 0.000 | 0.000 |
| △R^2^ | 0.002 | 0.063 | 0.001 | 0.017 | 0.000 | 0.000 |
| F | 1.130 | 44.246*** | 0.385 | 11.115*** | 0.277 | 0.262 |
| △F | 1.130 | 302.485*** | 0.385 | 75.456*** | 0.277 | 0.173 |

BMI, body mass index; CRC, colorectal cancer; BBPS, Boston Bowel Preparation scale.

**P* < 0.05，***P* < 0.01，****P* < 0.001

|  | Cecum +ascending colon | | Hepatic flexure | | Transverse colon | |
| --- | --- | --- | --- | --- | --- | --- |
|  | M13 | M14 | M15 | M16 | M17 | M18 |
| Age | 0.000 | 0.000 | 0.013 | 0.013 | 0.002 | 0.002 |
| Sex | 0.008 | 0.008 | -0.002 | -0.002 | 0.014 | 0.015 |
| Family history of CRC | 0.003 | 0.003 | 0.004 | 0.004 | 0.038* | 0.038* |
| BMI | 0.021 | 0.021 | 0.009 | 0.009 | 0.011 | 0.011 |
| Withdrawal time | -0.009 | -0.009 | 0.006 | 0.006 | 0.007 | 0.007 |
| BBPS | 0.073*** | 0.073*** | 0.060*** | 0.060*** | 0.070*** | 0.071*** |
| Deep-sedated colonoscopy |  | 0.016 |  | 0.003 |  | 0.019 |
| R^2^ | 0.006 | 0.006 | 0.004 | 0.004 | 0.007 | 0.007 |
| △R^2^ | 0.006 | 0.000 | 0.004 | 0.000 | 0.007 | 0.000 |
| F | 4.450*** | 3.988*** | 3.002** | 2.579* | 5.068*** | 4.590*** |
| △F | 4.450*** | 1.216 | 3.002** | 0.043 | 5.068*** | 1.715 |

Supplementary Table S2. Linear regression of adenomas detected in different colorectal segments

BMI, body mass index; CRC, colorectal cancer; BBPS, Boston Bowel Preparation scale.

**P* < 0.05，***P* < 0.01，****P* < 0.001

|  | Splenic flexure | | Descending colon | | Sigmoid colon +Rectum | |
| --- | --- | --- | --- | --- | --- | --- |
|  | M19 | M20 | M21 | M22 | M23 | M24 |
| Age | -0.019 | -0.018 | 0.010 | 0.009 | -0.015 | -0.015 |
| Sex | -0.004 | -0.003 | -0.006 | -0.006 | 0.000 | 0.001 |
| Family history of CRC | -0.004 | -0.003 | -0.012 | -0.013 | -0.007 | -0.007 |
| BMI | 0.017 | 0.017 | -0.025 | -0.025 | 0.006 | 0.006 |
| Withdrawal time | -0.014 | -0.014 | -0.003 | -0.003 | 0.019 | 0.019 |
| BBPS | 0.056*** | 0.057*** | 0.031* | 0.031* | 0.103*** | 0.103*** |
| Deep-sedated colonoscopy |  | 0.050*** |  | -0.004 |  | 0.012 |
| R^2^ | 0.004 | 0.006 | 0.002 | 0.002 | 0.011 | 0.011 |
| △R^2^ | 0.004 | 0.002 | 0.002 | 0.000 | 0.011 | 0.000 |
| F | 2.960** | 4.151*** | 1.433 | 1.238 | 8.389*** | 7.290*** |
| △F | 2.960** | 11.256*** | 1.433 | 0.069 | 8.389*** | 0.703 |

BMI, body mass index; CRC, colorectal cancer; BBPS, Boston Bowel Preparation scale.

**P* < 0.05，***P* < 0.01，****P* < 0.001
